# Impact of COVID-19 Pandemic on the Clinical Follow-Up of Patients Living with HIV in Chad: A Retrospective Monocentric Investigation

**DOI:** 10.4269/ajtmh.23-0384

**Published:** 2023-12-26

**Authors:** Marie Glenet, Guy Rodrigue Takoudjou Dzomo, Christian Nguemadjita, Nadia Djimera, Anne-Laure Lebreil, Yohan N’Guyen, Laurent Andreoletti

**Affiliations:** ^1^ Laboratory of Virology, CardioVir UMR-S 1320 INSERM, University of Reims Champagne Ardenne, Reims, France;; ^2^ Infectious Diseases Department, CHU Walia, Hôpital Le Bon Samaritain, N’Djamena, Chad;; ^3^ Infectious Diseases and Internal Medicine Department, CHU Reims, Hôpital Robert Debré, Reims, France;; ^4^ Virology Department, CHU Reims, Hôpital Robert Debré, Reims, France

## Abstract

The impact of the coronavirus disease 2019 (COVID-19) pandemic on the clinical follow-up of people living with HIV (PLWH) remains poorly documented in Sahelian Africa. We conducted a monocentric retrospective investigation of the outcomes (loss to follow-up [LTFU], transferred, or dead) among a cohort of PLWH receiving antiretroviral treatment (ART) in N’djamena, Chad (December 2019–December 2022). The incidence of LTFU was found to be higher in 2020 than in 2022 (*P* > 10^−4^), with increases of incidence of LTFU in the first trimester of 2020 before identified severe acute respiratory syndrome coronavirus 2 (SARS–CoV-2) infection cases in Chad. The all-cause mortality was low and did not appear to be influenced by SARS–CoV-2 infection waves. Our data reveal a concerning trend of significantly increased LTFU among PLWH receiving ART during the COVID-19 pandemic. Our findings indicate that it is crucial to provide accurate information to ensure the continuity of care for PLWH during a sanitary crisis in Sahelian Africa.

The spread of severe acute respiratory syndrome coronavirus 2 (SARS–CoV-2), responsible for the coronavirus disease 2019 (COVID-19) pandemic, has had a significant impact on access to health facilities in developed countries.^1^ However, some emergency healthcare pathways have managed to remain intact.^2^ Furthermore, studies have shown that the COVID-19 pandemic also affected access to HIV services in African countries, including South Africa.^3^ However, the impact on HIV services in developing countries with less robust healthcare systems has not been thoroughly investigated. Currently, there is a lack of available data in Chad, where we previously observed that stock-outs of antiretroviral treatment (ART) were associated with an increase in loss to follow-up (LTFU) among people living with HIV (PLWH).^4^ In this study, we retrospectively examined the impact of the COVID-19 pandemic on LTFU and mortality rates in a cohort of PLWH receiving ART despite the absence of ART stock-outs in “Le Bon Samaritain” Hospital, which is located in a particularly poor area of N’Djamena, Chad.

All 1,645 PLWH (1,107 females [63.7%]; median age, 33 [1–75] years) receiving ART and actively followed up in Le Bon Samaritain Hospital (N’Djamena, Chad)^4^ on December 31, 2019 were included. The study gathered data on the endpoints, including LTFU, transfer to another facility, or death, from the hospital register on a quarterly basis between December 31, 2019 and December 31, 2022.

Loss to follow-up was defined as cases in which PLWH were no longer actively being monitored but were not reported to have transferred to another facility or to have died. The PLWH who returned to care after being initially LTFU were excluded from the study cohort when they resumed care. The term “death” encompassed all-cause mortality and was not limited to mortality induced by COVID-19 specifically. No active investigation was performed to confirm that PLWH who were LTFU were in fact transferred or dead.

During the study period, peaks of SARS–CoV-2 genome detection in upper respiratory samples were evidenced in Chad during the first wave of the COVID-19 pandemic in May 2020, followed by a second larger wave from December 2020 to March 2021 with a peak in early January 2021 and a third wave peaking in January 2022, according to the Chadian national SARS-CoV-2 molecular survey.^5^ No ART stock-outs were reported during the study period according to Le Bon Samaritain Hospital data.

Cumulative incidence graphs were built, and statistical analyses were performed using GraphPad Prism 7 (Boston, MA) and SAS version 9.4 (SAS Institute Inc., Cary, NC). Comparison of cumulative incidence was performed using a log rank (Mantel-Cox) test. A *P* value < 0.05 was considered significant.

An annual stratified analysis was conducted on PLWH receiving ART, revealing a 20% decrease in the number of PLWH accessing ART in the outpatient setting during the first year of the COVID-19 pandemic (*N* = 1,645 compared with *N* = 1,249). This decline was attributed to LTFU, with 409 individuals classified as LTFU compared with 22 deaths and 8 transfers (Figure 1A). Notably, 14 patients who were previously LTFU returned to care during the study period and were subsequently excluded (data not shown). To understand the dynamics of LTFU throughout the study period, cumulative incidence plots were generated for the endpoints of LTFU, transfer, or death (Figure 1B). The incidence of LTFU started to increase in the first trimester of 2020, preceding the peak detection of SARS–CoV-2 genomes in Chad (Figure 1B). Furthermore, the incidence of LTFU was higher in 2020 than in 2021 or 2022 (*P* < 0.0001) (Figure 1C). The overall all-cause mortality rate remained relatively low, with 36 of 1,645 PLWH experiencing mortality (2.4%; Figure 1A). The increase in mortality incidence was less pronounced than LTFU and did not appear to be influenced by the peaks of SARS–CoV-2 genome detection in Chad (Figure 1B). In addition, the incidence of referral to another center as an endpoint appeared to remain consistent throughout the study period (Figure 1A and B).

**Figure 1.**
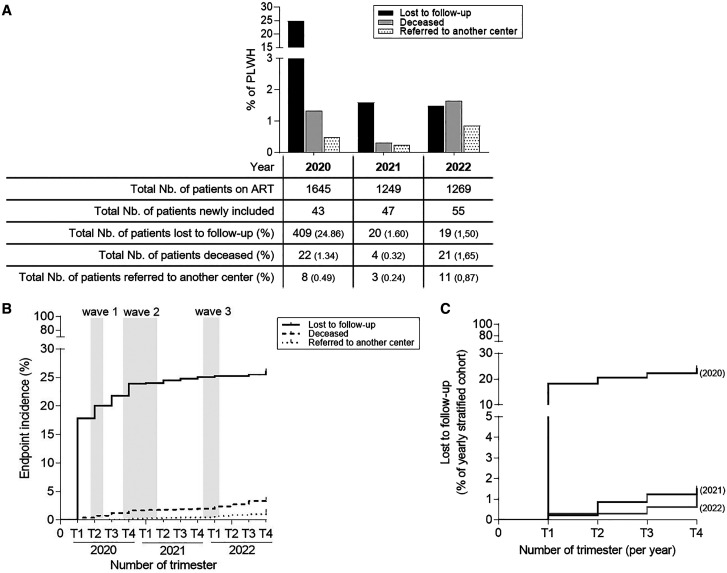
Impact of coronavirus disease 2019 (COVID-19) pandemic (2020–2022) on a cohort of Chadian outpatients receiving antiretroviral treatment in Le Bon Samaritain Hospital (N’Djamena, Chad) between December 31, 2019 and December 31, 2022. (**A**) Yearly stratified analysis of the included patients according to their endpoints at the end of years 2020, 2021, and 2022. (**B**) Cumulative incidence graphs of each endpoint among patients living with HIV (PLWH) during the study period. Each endpoint incidence is expressed as a percentage of the whole cohort. Gray areas show severe acute respiratory syndrome coronavirus 2 (SARS–CoV-2) infection waves in Chad. (**C**) Comparison of the incidence of attrition/loss to follow-up between years 2020 and 2022. The attrition incidence is expressed as a percentage of the whole cohort. ART = antiretroviral treatment; Nb. = significate number; T = trimester.

In the present report, we aimed to shed light on the effects of the COVID-19 pandemic on Chadian outpatients receiving ART, enabling us to develop strategies to mitigate any negative consequences and improve patient outcomes in case of further pandemics or a sanitary crisis. Within a well–clinically monitored monocentric cohort of PLWH, we compared the dynamics of our study cohort, focusing specifically on the incidence of attrition/LTFU and mortality rates between the years of the study’s COVID-19 pandemic period (Figure 1). Notably, we observed that the onset of the COVID-19 epidemic resulted in a 20% reduction in the cohort of PLWH receiving ART and being clinically monitored at Le Bon Samaritain Hospital in N’Djamena, Chad (Figure 1A). This decrease occurred during the first trimester of 2020, prior to the detection of SARS–CoV-2 genomes in Chadian patients, and was attributed to LTFU of significant numbers of clinically monitored HIV-infected patients (PLWH).

To try exploring the potential reasons of LTFU among PLWH, we compared LTFU subgroup patients with available data concerning WHO stage and CD4 cell counts with those actively followed up with available data concerning WHO stage and CD4 cells count (Table 1). We did not identify significant differences between these two patient subgroups, but it seems to be a trend toward a higher WHO stage and a lower T-CD4 count in LTFU patients, suggesting that identified LTFU patients might be dead. However, this observed decline in PLWH accessing ART could be explained mainly by the disruption of healthcare systems caused by COVID-19 or the fear of infection at healthcare facilities, which posed a threat to accessing HIV treatment services.^5^^,^^6^ Our observed consistent incidence of the “referral to another center” endpoint throughout the study period indicated that healthcare facilities were not overcrowded, and the coordination between them was not significantly affected by the COVID-19 pandemic. It is worth noting that there were no care disruptions, such as healthcare provider withdrawal or transportation system shutdowns, during the study period, except for a curfew in place during nighttime from April 2, 2020 to March 11, 2021.^6^^–^^9^ Thus, these findings suggest that the fear of SARS–CoV-2 infection could result in the significant and early LTFU observed. Indeed, in Africa, PLWH have been reported to avoid hospitals because of concerns about exposure to the coronavirus, and misinformation about the virus has exacerbated this fear.^10^^,^^11^

**Table 1:** Comparison of demographic, biological, and clinical characteristics between LTFU and actively followed patient subgroups with available data concerning WHO stage and CD4 cell counts

Characteristic	LTFU (*N* = 45)	Actively followed (*N* = 1,110)	*P* value
Females (%)	26 (57.7)	651 (58.6)	0.9*
Age in years, median (min-max)	30 (5–67)	31 (1–80)	0.62†
CD4 median (min-max)	217 (25–615)	253 (1–2,443)	0.08†
WHO stage median (min-max)	3 (1–4)	2 (1–4)	0.053†

LTFU = loss to follow-up.

* Statistical comparative analysis was performed using Pearson χ^2^ test.

† Statistical comparative analysis was performed using Mann-Whitney *U* test.

Although SARS–CoV-2 infection could be associated with an increased risk of mortality among PLWH, the confirmed all-cause mortality in our study cohort remained low (2.4%) during the COVID-19 pandemic period. This could be partly explained by the young median age of study PLWH (median age of 33 years), which may have significantly contributed to a lower COVID-19–induced mortality. However, recently published studies in similar young HIV-infected populations described an increased risk of mortality by chronic cardiovascular pathologies, diabetes, malaria, or tuberculosis.^12^^,^^13^ Moreover, by contrast to these published reports concerning PLWH surveys in Africa during the COVID-19 pandemic, we did not observe any significant interruption of ART availability related to ART drug stock-outs promoting therapeutic and clinical failures and subsequently increasing mortality rates in such HIV-infected patients. However, our retrospective investigation is not fully representative because we investigated data from only one well-clinically monitored monocentric cohort of PLWH in N’Djamena, Chad. Another key limitation of our retrospective investigation is the lack of active investigation to confirm whether PLWH who were LTFU had indeed transferred or died. To this end, it would have been helpful to collect phone numbers at the time of cohort inclusion for each study patient, therefore allowing monitoring of LTFU patients by calling them or sending them phone messages. Whatever, it is known that LTFU is associated with poorer outcomes, and only a small proportion of patients who were LTFU returned to care.^14^ Efforts should be intensified to locate and reengage PLWH who have been LTFU, not only in our monocentric cohort but also in other cohorts in Chad and across sub-Saharan African countries.^15^

In conclusion, our retrospective epidemiological findings reveal a concerning trend of significantly increased LTFU among Chadian individuals living with HIV (PLWH) who were receiving ART during the COVID-19 pandemic. This unexpected observation can be attributed to the heightened fear of SARS–CoV-2 infection within African hospital settings. It is crucial to address these concerns and provide accurate information to ensure continuity of care for PLWH during times of sanitary crisis in Sahelian Africa countries.

## References

[1] Moynihan R , 2021. Impact of COVID-19 pandemic on utilisation of healthcare services: a systematic review. BMJ Open 11: e045343. 10.1136/bmjopen-2020-045343 PMC7969768 33727273

[2] Demotier S Gornet M Belli A Huguenin A N’Guyen Y, 2023. Malaria diagnosis in an emergency department before and after the COVID-19 pandemic: a retrospective study. Trans R Soc Trop Med Hyg 117: 64–66. 35903001 10.1093/trstmh/trac073 PMC9384599

[3] Abdool Karim Q Baxter C, 2022. COVID-19: impact on the HIV and tuberculosis response, service delivery, and research in South Africa. Curr HIV/AIDS Rep 19: 46–53. 35064888 10.1007/s11904-021-00588-5 PMC8783194

[4] Djarma O Nguyen Y Renois F Djimassal A Banisadr F Andreoletti L, 2014. Continuous free access to HAART could be one of the potential factors impacting on loss to follow-up in HAART-eligible patients living in a resource-limited setting: N’djamena, Chad. Trans R Soc Trop Med Hyg 108: 735–738. 25163753 10.1093/trstmh/tru130

[5] World Health Organization *Chad: WHO Coronavirus Disease (COVID-19) Dashboard With Vaccination Data*. Available at: https://covid19.who.int/region/afro/country/td. Accessed June 8, 2023.

[6] *(COVID-19) Le Tchad Instaure un Couvre-Feu à Compter de ce Jeudi_French.news.cn* Beijing, China: *xinhuanews*. Available at: http://french.xinhuanet.com/2020-04/02/c_138941643.htm. Accessed May 30, 2023.

[7] Dabesne M, 2021. Tchad: Le couvre-feu prorogé pour deux semaines à N’Djaména et dans quelques provinces. *Tchadinfos.com* . Available at: https://tchadinfos.com/oktchad-le-couvre-feu-proroge-pour-deux-semaines-a-ndjamena-et-dans-quelques-provinces/. Accessed May 30, 2023.

[8] Tchad: le couvre-feu prolongé de deux semaines, de 21h à 5h *Alwihda Info – Actualités TCHAD, Afrique, International*. Available at: https://www.alwihdainfo.com/Tchad-le-couvre-feu-prolonge-de-deux-semaines-de-21h-a-5h_a100411.html. Accessed May 30, 2023.

[9] *COVID-19 Pandemic - Chad* Available at: https://global-monitoring.com/gm/page/events/epidemic-0002027.1DbrZymYF1IF.html?lang=en. Accessed June 8, 2023.

[10] Aristide C Okello S Bwana M Siedner MJ Peck RN, 2020. Learning from people with HIV: their insights are critical to our response to the intersecting COVID-19 and HIV pandemics in Africa. AIDS Behav 24: 3295–3298. 32607916 10.1007/s10461-020-02955-6 PMC7325644

[11] Nachega JB , 2021. Minimizing the impact of the triple burden of COVID-19, tuberculosis and HIV on health services in sub-Saharan Africa. Int J Infect Dis 113: S16–S21. 33757874 10.1016/j.ijid.2021.03.038 PMC7980520

[12] Ssentongo P Heilbrunn ES Ssentongo AE Advani S Chinchilli VM Nunez JJ Du P, 2021. Epidemiology and outcomes of COVID-19 in HIV-infected individuals: a systematic review and meta-analysis. Sci Rep 11: 6283. 33737527 10.1038/s41598-021-85359-3 PMC7973415

[13] Jassat W , 2021. Risk factors for COVID-19-related in-hospital mortality in a high HIV and tuberculosis prevalence setting in South Africa: a cohort study. Lancet HIV 8: e554–e567. 34363789 10.1016/S2352-3018(21)00151-X PMC8336996

[14] Martinez-Guerra BA Valdez-Ventura R Caro-Vega Y Sierra-Madero JG Crabrree-Ramírez BE, 2022. Gaps in the continuum of care in HIV-positive adults and the need for caution in those returning to care after loss to follow-up. AIDS Care 35: 1604–1611. 36529962 10.1080/09540121.2022.2150139

[15] Ballif M , 2022. Tracing people living with human immunodeficiency virus who are lost to follow-up at antiretroviral therapy programs in southern Africa: a sampling-based cohort study in 6 countries. Clin Infect Dis 74: 171–179. 33993219 10.1093/cid/ciab428 PMC8800181

